# Habitat Suitability and Conflict Zone Mapping for the Blue Bull (*Boselaphus tragocamelus*) across Nepal

**DOI:** 10.3390/ani13050937

**Published:** 2023-03-05

**Authors:** Bijaya Dhami, Arjun Bhusal, Binaya Adhikari, Mahamad Sayab Miya, Surya Kumar Maharjan, Dinesh Neupane, Hari Adhikari

**Affiliations:** 1 Department of Biological Sciences, University of Alberta, 116 St & 85 Ave, Edmonton, AB T6G 1H9, Canada; 2 IUCN/SSC Deer Specialist Group, 1196 Gland, Switzerland; 3 School of Forestry and Natural Resource Management, Institute of Forestry, Tribhuvan University, Kathmandu 44600, Nepal; 4 Green Governance Nepal, Kathmandu 44600, Nepal; 5 Pokhara Zoological Park and Wildlife Rescue Center, Kaski 33700, Nepal; 6 Institute of Forestry Pokhara Campus, Tribhuvan University, Pokhara 33700, Nepal; 7 Institute of Forestry Hetauda Campus, Tribhuvan University, Hetauda 44107, Nepal; 8 Southeast Asia Biodiversity Research Institute, Chinese Academy of Sciences and Center for Integrative Conservation, Xishuangbanna Tropical Botanical Garden, Chinese Academy of Sciences, Mengla, Kunming 666303, China; 9 Resources Himalaya Foundation, Naya Bato, Sanepa, Lalitpur 44600, Nepal; 10 Department of Geosciences and Geography, University of Helsinki, P.O. Box 68, 00014 Helsinki, Finland

**Keywords:** ensemble modelling, human–Blue bull conflict, species distribution modelling, environmental changes, crop raiding, retaliatory killing

## Abstract

**Simple Summary:**

Rapidly changing environmental conditions can alter the spatial distribution of flora and fauna. This study aimed to understand the influence of environmental variables on the Blue bull’s distribution and identify potential conflict zones in Nepal. Using ensemble modeling, the habitat suitability analysis of the Blue bull was performed by selecting 15 ecologically significant environmental variables and employing ten species distribution modeling algorithms. Random Forest, Maxent, and Generalized linear models showed the highest mean true skill statistics scores and were further analyzed. The study found that 15.26% of Nepal, or 22,462.57 km^2^, is suitable for the Blue bull, and the environmental variables contributing to the distribution of the Blue bull were slope, precipitation seasonality, and distance to the road. Furthermore, 45% of the predicted suitable habitats overlap with agricultural land, highlighting the potential for human–Blue bull conflicts. Therefore, this study recommends implementing appropriate conflict mitigation measures, such as cooperatively guarding crops, changing cropping patterns, using repellents, fencing, translocation, physical barriers, and sterilization. This study establishes a baseline for suitable habitats for the Blue bull and identifies potential conflict zones in Nepal, emphasizing the need for conservation initiatives inside and outside protected areas.

**Abstract:**

Rapidly changing environmental conditions (bioclimatic, anthropogenic, topographic, and vegetation-related variables) are likely to alter the spatial distribution of flora and fauna. To understand the influence of environmental variables on the Blue bull’s distribution and to identify potential conflict zones, the habitat suitability analysis of the Blue bull was performed using ensemble modeling. We modelled the distribution of the Blue bull using an extensive database on the current distribution of the Blue bull and selected 15 ecologically significant environmental variables. We used ten species distribution modeling algorithms available in the BIOMOD2 R package. Among the ten algorithms, the Random Forest, Maxent, and Generalized linear model had the highest mean true skill statistics scores, ensuring better model performance, and were considered for further analysis. We found that 22,462.57 km^2^ (15.26%) of Nepal is suitable for the Blue bull. Slope, precipitation seasonality, and distance to the road are the environmental variables contributing the most to the distribution of Blue bull. Of the total predicted suitable habitats, 86% lies outside protected areas and 55% overlaps with agricultural land. Thus, we recommend that the future conservation initiatives including appropriate conflict mitigation measures should be prioritized equally in both protected areas and outside protected areas to ensure the species’ survival in the region.

## 1. Introduction

The Blue bull (*Boselaphus tragocamelus*), commonly known as ‘Nilgai’, is Asia’s largest antelope, a member of the Bovidae family listed as least concern globally [1,2]. However, in Nepal, it is categorized as a nationally vulnerable species due to its declining population from suspected poaching, retaliatory killing, and habitat loss [3]. It is the only sexually dimorphic ungulate of huge stature and distinctive color in the genus *Boselaphus* [4]. This species has a wide distribution in the lowlands of Nepal and India, extending into the borders of Pakistan, and is now extinct from Bangladesh [1]. The species has already been introduced in Texas, Mexico, South Africa, and Italy [4]. 

In Nepal, it is distributed in protected and non-protected areas of the southern plains called ‘Terai’ [5] across habitat types like grassy steppe forests, scrub areas, flood plains, dry deciduous forests, riverine forests, and the agricultural regions [6]. It appears to thrive in Koshi Tappu Wildlife Reserve in the east, Parsa National Park in the middle, and Shuklaphanta and Bardia National Parks in the west [5,7]. It prefers various habitat types, including hillsides, arid areas, grassy steppe forests, scrub areas, flood plains, dry deciduous forests, riverine forests, and agricultural areas [6].

Due to its preference and dependency on agricultural land, it causes substantial financial losses and is now considered a pest in India and Nepal [4]. As a result, retaliatory killing is prevalent, causing a drastic decline in the species’ population in Nepal [6]. In addition, its habitat is being fragmented and degraded due to increased human settlement, infrastructure development, overgrazing, and agricultural expansion, further threatening the country’s population [3].

Mapping and predicting potentially suitable habitats of threatened and conflict-creating taxa is critically essential from the monitoring and management perspective [8]. The first step toward effective wildlife conservation is habitat assessment [9]. It offers information on the quality and quantity of the habitat available for targeted species [10]. Habitat modeling is primarily used in conservation planning to estimate the geographical distribution of appropriate habitats for species of interest in a landscape [11]. 

Species distribution modeling (SDM), often referred to as ecological niche modeling, builds a species–environment relationship to explain and forecast the likely distribution of a species [12,13,14]. SDM can be used as a conservation planning approach for threatened species by determining the species distribution range and ecological niche [15]. Due to extensive data and multifaceted associations between species and ecological variables, the scope of machine learning methods such as SDM has increased to solve the problem of ecologists and statisticians [12]. Besides, SDM helps to envisage the effects of climate change on species, which is essential to achieve the conservation goals of awareness of the species distribution [16,17]. Several modeling techniques are assembled through the ensemble method in SDM to improve the projecting performance [10]. 

However, a science-based conservation plan in Nepal needs to be improved to address these issues. Identifying suitable habitats across the region and mapping potential conflict zones help guide the conservation action for the long-term survival of this species. This study tried to explore the current suitable habitat and conflict zones across Nepal by applying the ensemble model. In this study, (a) we modeled the distribution of the Blue bull in Nepal based on bio-climatic, bio-physical, anthropogenic, and topographic variables using an ensemble modeling approach; (b) identified key factors affecting the Blue bull distribution; and (c) identified the potential conflict zones by combining the habitat suitability map with the land use and land cover map. We believe this study will be instrumental in prioritizing conflict zones and formulating proper conflict mitigation strategies to ensure the species’ long-term conservation.

## 2. Materials and Methods

### 2.1. Study Area

**Figure 1:**
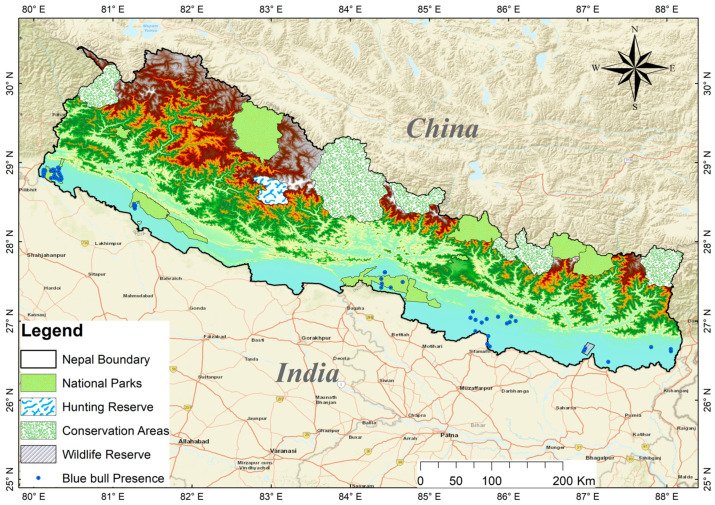
Map of Nepal showing the location of the protected areas of different categories. The blue dots show the current distribution locations of the Blue bull across Nepal.

Nepal is a mountainous nation in South Asia. It covers an area of 147,516 km^2^ and is located between the latitudes of 26°22′–30°27′ N and the longitudes of 80°04′–88°12′ E. It is endowed with extensive biodiversity due to its variable climate and topography along a steep altitudinal gradient from 60 to 8848 m a.s.l. [18,19]. Nepal can be divided into three main physiographic zones: the lowland (Terai and Siwalik), the mid-hills, and the high mountains [20]. The climate is usually mild, with dry winters and rainy summers [21]. Its mean annual precipitation is 1768 mm and the mean annual temperature is 18 °C [22]. The Blue bull in Nepal is restricted to alluvial flood plains in the southern lowlands [5]. The southern lowlands of Nepal are home to seven protected areas, including Shuklaphanta National Park (SNP), Bardia National Park (BNP), Banke National Park (BaNP), Krishnasar Conservation Area (KCA), Chitwan National Park (CNP), Parsa National Park (PNP), and Koshi Tappu Wildlife Reserve (KTWR) (Figure 1).

### 2.2. Data Collection

#### 2.2.1. Blue Bull Presence Data

The occurrence points of the Blue bull were obtained primarily from field-based surveys conducted between 2018 and 2021. The periodic data from the Department of National Park and Wildlife Conservation (DNPWC) and the Division Forest Offices (DFOs) obtained through personal communications were used as supplementary data. Direct observation, pellet droppings, and hoofmarks were used to confirm the Blue bull’s presence in an area. In total, 179 occurrence points of the species were collected from the distribution range. The occurrence data were cleaned by removing duplicates and locations that tend to fall beyond the species’ reported distribution area. We chose only one presence point if multiple presence points are available within a grid of 1 × 1 km using a SpThin package in R [23], given that environmental factors with a spatial resolution of 1 × 1 km were used to predict species habitat refugia.

#### 2.2.2. Environmental Variables 

We used a combination of bioclimatic, anthropogenic, topographic, and vegetation-related variables for the habitat suitability model. We tried to include all the significant predictor variables because variable selection is an essential step in species distribution modeling (SDM) [24]. We compiled 33 variables (Table 1) crucial for the Blue bull’s habitat suitability modeling [25]. 

Due to their ecological significance and ability to define annual patterns, seasonality, and extremes of temperature and precipitation, bioclimatic variables are widely employed in spatial modelling [26,27]. To retrieve 19 bioclimatic variables, WorldClim-Global Climate Data (https://www.worldclim.org/data/worldclim21.html) was used [28]. These data were obtained in a grid format with a spatial resolution of 1 × 1 km.

The anthropogenic variables used in our model include distance to a human path, distance to roads, distance to settlements, human population density, livestock density, and land use land cover data. The data on the paths, roads, and buildings were extracted from Geofabrik’s website [29]. Data on settlements were assessed from the Nepalese Department of Survey, and a distance raster file was created using ArcGIS10.8.1 [30]. The data on land use and land cover change was accessed from the ICIMOD [31]. Similarly, human population density and livestock density data were accessed through the Humanitarian Data Exchange Dataset [32] and Open Data Nepal [33].

Topographic variables were included as predictor variables in our model. Using ArcMap 10.8.1 [30], elevation, aspect, and slope data were extracted from a Digital Elevation Model (DEM) with a 1 km spatial resolution that was downloaded from the US Geological Survey database [34]. Water source shapefiles were collected from the Geobabrik website [29] and transformed using ArcMap 10.8.1 [30] into a distance raster file.

The following four vegetation-related variables were collected for this study: forest cover, minimum Enhanced Vegetation Index (EVI), mean EVI, and maximum EVI. Forest cover was extracted from Earth engine partner Appspot [35]. Moderate Resolution Imaging Spectroradiometer (MODIS) [34] was used to download EVI time-series data. The Savitzky-Golay filter was used to smooth the data in the TIMESAT algorithm [36]. This technique helped to reduce cloud cover in the surroundings and facilitated image visualization. Later, the average values of overall indices were calculated to obtain the final index of EVI.

Next, the multi-collinearity test was performed among 33 environmental variables, and variables with correlation coefficients > 0.7 and variance inflation factor > 5 were removed to avoid multi-collinearity [37]. After the multi-collinearity test, 15 predictor variables were left and used for habitat suitability modeling for the Blue bull in Nepal (Table 1).

### 2.3. Data Analysis

#### 2.3.1. Blue Bull Habitat Suitability Modeling

We used the overview, data, model, assessment, and prediction method described by Zurell et al. (2020) [38] to develop habitat suitability models for the Blue bull in Nepal. Given its improved predictive accuracy [10], recent SDM exercises praise combining multiple models created using various modeling techniques into an ensemble map [39]. Therefore, we also developed habitat suitability models for the Blue bull using an ensemble modeling approach. In order to generate ensemble models in R, the BIOMOD2 package [31] was used (R Development Core Team 2020). Ten algorithms, including the artificial neural network (ANN), the classification tree analysis (CTA), the flexible discriminant analysis (FDA), the generalized additive model (GAM), the generalized boosting model (GBM), the generalized linear model (GLM), the multiple adaptive regression splines (MARS), the maximum entropy (MAXENT), the random forest (RF), and the surface range envelope (SRE) were used to create the ensemble models. We created 10,000 random pseudo-absence points, as postulated by Barbet-Massin et al. (2012) [40], by assigning equal weight to the presence and pseudo-absence datasets and repeating the pseudo-absence generation three times to minimize random bias. The Blue bull’s presence and pseudo-absence data were split into training (70%) and testing (30%) data sets. Our modeling approach included ten algorithms, three pseudo-absence selection runs, and three evaluation runs. This yielded a total of 90 model runs. The receiver operating characteristics (ROC) curve, referred to as area under the curve (AUC) [41], and true skill statistics (TSS) [42] are two independent techniques that are frequently used to evaluate the accuracy of predictive distribution models [13]. Despite its widespread use as a model evaluation metric, AUC has been rebuked for its limitations [41]. Therefore, the TSS evaluation criterion (−1 to +1) was used to assess our model’s predictive performance. If the TSS value is +1, the model is considered perfect, whereas a TSS value between 0.7 and 0.9 indicates a good model [13,42]. We used the weighted mean approach to build an ensemble model from all models with a TSS value greater than 0.6 [43]. Three models (GMB, MaxEnt, and RF) have TSS value greater than 0.6 and hence were used to develop the weighted mean ensemble approach.

#### 2.3.2. Potential Conflict Zones Mapping

To map potential human–Blue bull conflict zones, we overlaid the suitable habitats over the cropland extracted from the LULC map downloaded from the ICIMOD website [44]. The overlapped area was then extracted.

## 3. Results

### 3.1. Predicted Suitable Habitats for the Blue Bull

**Figure 2:**
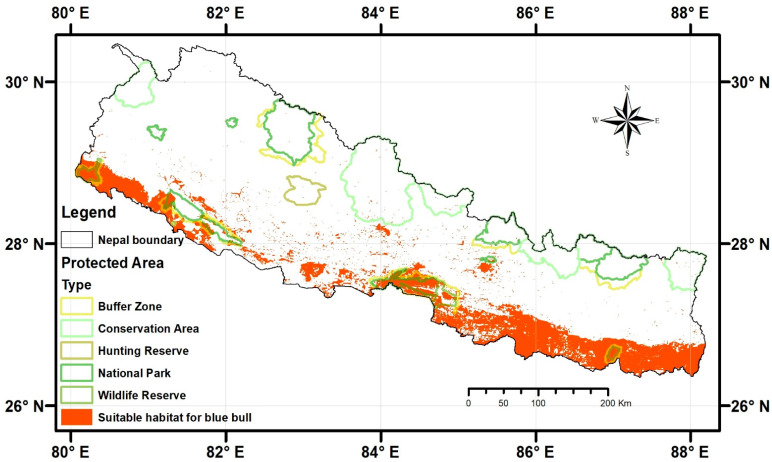
Predicted suitable habitats (orange color) for the Blue bull in Nepal. The protected areas of different categories across Nepal are also shown.

The habitat suitability map generated using an ensemble modeling approach showed that 22,462.57 km^2^ (15.26%) of Nepal is suitable for the Blue bull (Figure 2).

Of the total suitable habitat (22,462.57 km^2^), only 14.27% (3204.35 km^2^) falls inside the protected area management system, while 85.73% (19,257.16 km^2^) is located outside the protected area system. The buffer zone of Koshi Tappu Wildlife Reserve has the highest proportion of suitable area (100%), followed by Shukhlaphanta Buffer Zone (94%), Chitwan National Park (91%), and Shuklaphanta National Park (89%), out of all management systems. Protected areas, mainly covering the mid-hill area, had the lowest proportion of suitable habitat. Out of the total suitable habitat area included inside the protected areas, Chitwan National Park contained the highest proportion of suitable habitats (27%), followed by its buffer zone (16%, see Table 2 for more details).

We also found that most of the predicted suitable habitat occurs in the Terai and the Siwalik regions of Eastern and Central Nepal (Figure 2).

### 3.2. Factors Affecting the Blue Bull’s Distribution in Nepal

Of the 15 predictor variables used to model the suitable habitats for the Blue bull, slope contributed the most to the model, followed by precipitation seasonality (see Table 3 for detail).

**Figure 3:**
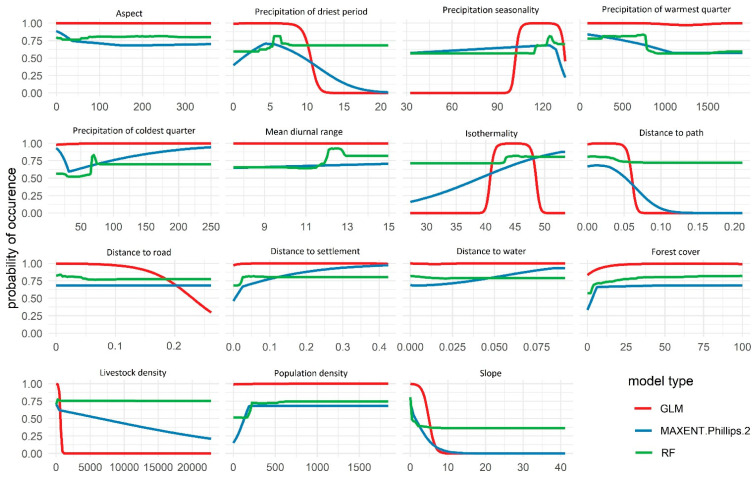
Response curves indicating the effects of different environmental variables on the habitat suitability of the Blue bull in Nepal.

The response curves of GLM, MaxEnt, and RF indicate that the probability of occurrence of the Blue bull decreases with an increase in slope; more specifically, areas with slope > 10 degrees are unsuitable for the Blue bull, whereas it peaks in areas with precipitation seasonality between 95 and 130 (Figure 3). Furthermore, the suitability decreases with an increase in distance to road (Figure 3).

### 3.3. Models Accuracy in Predicting the Current Suitable Habitat of the Blue Bull

**Figure 4:**
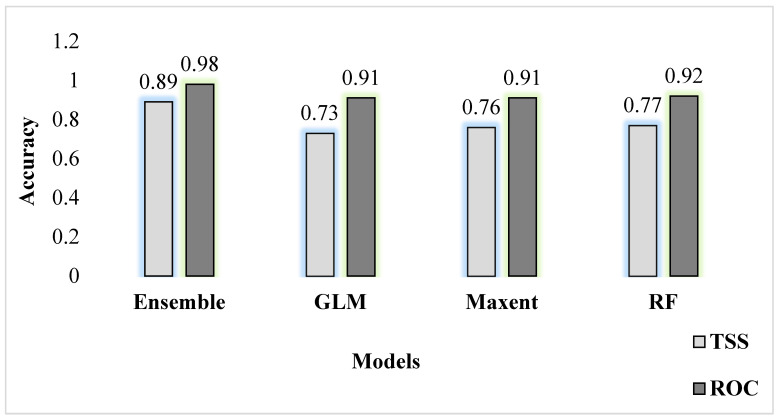
Performance predictions of various modeling approaches used to develop a current suitable habitat model based on true skill statistics.

Among the ten algorithms, RF, Maxent, and GBM had the highest mean TSS scores, ensuring better model performance. Regarding predictive performance, the ensemble model surpassed (0.89) single algorithm models based on TSS (Figure 4).

### 3.4. Potential Human–Blue Bull Conflict Zones

**Figure 5:**
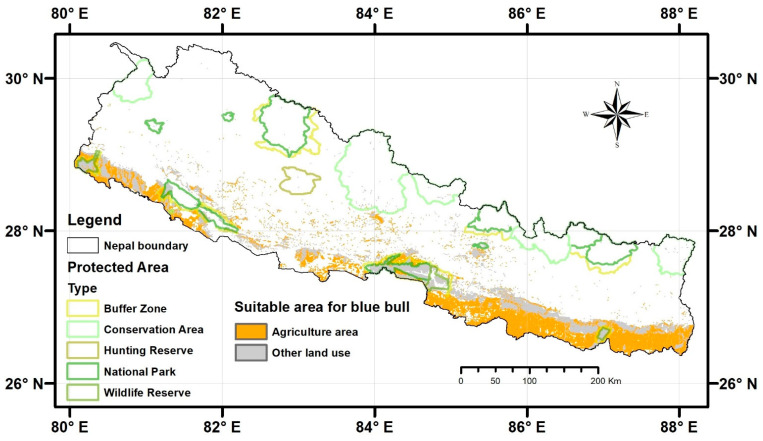
Map showing the overlap between suitable habitats of the Blue bull and different land use categories.

Upon layering the suitable habitat of the Blue bull over the LULC layer, we found that agricultural land overlaps with 54.8% of the total suitable area of the Blue bull and with 45.2% by other land use categories (forest 34%, shrubland 1%, grassland 2%, barren land 5%, water body 2%, and built-up areas 1%) (Figure 5).

## 4. Discussion

In this study, we modeled the potential distribution and mapped the potential conflict zones of the Blue bull in Nepal. We found that 22,462.57 km^2^ (15.26%) of Nepal is suitable for the Blue bull (Figure 2 and Table 2). Slope, precipitation seasonality, and distance to the road are the environmental variables contributing the most to the Blue bull habitat suitability models. Almost 55% of the predicted suitable habitats fall under agriculture land use, suggesting that these areas could be potential human–Blue bull conflict zones (Figure 5). Below we discuss the mechanisms likely to underlie the aforementioned findings.

### 4.1. Predicted Suitable Habitats for the Blue Bull

This study predicted that 22,462.57 km^2^ of Nepal is suitable for the Blue bull (Figure 2 and Table 2), which is 4200 km^2^ more than the area (18,213 km^2^) estimated by Jnawali et al. (2011) [3]. In the last decade, Nepal’s forest coverage, particularly outside the protected areas, increased from 37% to 44.74% [45,46], which might have contributed to this increase in the predicted suitable habitat for the Blue bull in Nepal. Besides, the predicted wider distribution of the Blue bull can be explained by its generalist nature. The Blue bull can survive in various habitats like arid areas, scrub, grassy plains, dry deciduous open forests, and agricultural areas [1]. 

Ref. [3] reported that the Blue bull is found in the protected areas (Chitwan National Park, Bardia National Park, Koshi Tappu Wildlife Reserve, Parsa National Park, and Shuklapanta National Park) as well as outside of them in Parsa, Rupandehi, Nawalparasi, Kailali, Kanchanpur, and Bardia districts. The presence of the Blue bull outside protected areas in Nepal was also documented by Ref. [47], who recorded 40 Blue bulls from grasslands and open forests of the Tinahu river of Rupandehi district; Ref. [7] reported 303 Blue bulls from Rupandehi; Ref. [48] reported 15 Blue bulls from Laljhadi Mohana Biological Corridor; Ref. [49] documented Blue bulls from the fragmented habitats of the Sarlahi, Rautahat, and Kamala River of Central Nepal; and Ref. [50] recently reported a Blue bull from the Jalthal forest of Jhapa in Eastern Nepal. In line with the findings of these studies, we also found that more than 85% of the predicted suitable habitats lie outside the protected areas. This suggests that the future conservation initiatives should not only prioritize protected areas (as most of the conservation initiatives in Nepal are) but also consider species conservation outside protected areas and establish corridors and connectivity among the forest patches outside of protected areas to ensure the species’ survival in the region.

### 4.2. Factors Affecting the Blue Bull’s Distribution in Nepal

We found that slope, precipitation seasonality, and distance to the road are the environmental variables contributing the most to the Blue bull habitat suitability models (Figure 3 and Table 3). More specifically, we found that areas with slope < 10 degrees are suitable for the Blue bull. This is in line with [51,52,53], which suggest that flat areas and the marginal slopes are preferred by the Blue bull. About precipitation seasonality, the probability of occurrence of the Blue bull was found to be maximum in the areas with precipitation seasonality ranging between 95 and 130, suggesting that eastern and central lowlands of Nepal that receive heavy rainfall during monsoon and little or no rain during other seasons are more suitable for the Blue bull [54]. This might have to do with the mixed feeding nature of the Blue bull, since it prefers to feed from various grass species [55], and precipitation seasonality favors the growth of mixed plant species and maintains seasonal grassland dynamics [56]. With the increased rainfall, the forest becomes dense, with little grassland area to support the low-density terrestrial herbivores [57]. Ref. [52] reported that open habitats are preferred by the Blue bull, which coincides with our findings that suggest that areas closer to the roads are more suitable for the Blue bull. Ref. [5] reported that despite having higher human disturbance near roads or human settlements, the Blue bull prefers to remain on the edge of the forests and agricultural land due to a higher species diversity in these regions. The Blue bull indeed has been reported to be more tolerant to human disturbances than other ungulates such as the Sambar and Chittal [53,58,59]. Moreover, research has revealed that the Blue bull tends to avoid dense forests [1,4,60] and to inhabit open grasslands, margins, and buffer zones with low-density forests [5] to reduce the risk of predation, since it can have better visibility of tigers in open and low-density habitats [61]. Such phenomena of avoiding dense forests could make them more likely to reside in the buffer zones and outside the protected areas, increasing the conflict with humans. 

Concerning precipitation seasonality, the probability of occurrence of the Blue bull was found to be maximum in areas with precipitation seasonality ranging between 95 and 130. The lowlands in Central and Eastern Nepal that receive heavy rainfall during monsoon and little or no rain during other seasons [54] hold higher suitability. Additionally, precipitation influences the growth of plant species and grassland dynamics [56]. The Blue bull is a mixed feeder that prefers to feed from various grass species [55]. As precipitation rates increase, the forest ecosystem experiences an increase in vegetative density, resulting in a reduction of grassland habitats. This restricted availability of grassland resources may limit the carrying capacity of low-density terrestrial herbivores within the ecosystem [57].

### 4.3. Potential Human–Blue Bull Conflict Zones

We found that almost 55% of the predicted suitable habitats overlap with agriculture land, suggesting that these areas could be potential human–Blue bull conflict zones (Figure 5). Such considerable overlap could be an indication of the phenomenon that the Blue bull tends to avoid dense forests [1,4,60] and to occupy open grasslands, edges, and buffer zones with low-density forests [5] to avoid predation risk. However, this might subsequently lead to increased human–Blue bull conflict. Indeed, human–Blue bull conflict, particularly crop damage, has been reported to cause substantial financial losses in India and Nepal. In response to such conflict, retaliatory killing is prevalent in India and Nepal [6]. Importantly, illegal hunting, electric fencing, poisoning, and habitat deterioration have become significant threats to the Blue bull in a human-dominated landscape [7]. Thus, proper conflict mitigation strategies should be adopted. In response to the ongoing human–Blue bull conflict, several mitigation measures have been employed by farmers. Fences, crop guards, night lights, drum beating, and dogs in the buffer zone of Bardia National Park [5], as well as firecrackers, trenches, electric fences, and scarecrows in Rupandehi [7] have been used frequently. Similarly, Refs. [62,63] have suggested that the cooperative guarding of crops during the harvesting season, changing cropping patterns, using (olfactory, acoustic, and visual) repellents, fencing, translocation, physical barriers, and sterilization could be beneficial to mitigate crop loss from the Blue bull and reduce the retaliatory killing. 

## 5. Conclusions

This study is the first of its kind to model the potential distribution and mapped the potential conflict zones of the Blue bull across Nepal. In this sense, it has established a baseline about the suitable habitats for the Blue bull and the potential human–Blue bull conflict zones in Nepal. We found that over 15% of the country’s land is suitable for the Blue bull, which is much higher than earlier estimates. We also found that the majority of the predicted suitable areas lies outside of the protected areas, which in turn suggests that the future conservation initiatives should not only prioritize protected areas but also consider species conservation outside of them. Similarly, establishing corridors and connectivity among the forest patches outside of protected areas could ensure the species’ survival in the region. We also found that the Blue bull prefers the flat lowlands of Eastern and Central Nepal with clear monsoon and dry season, and we confirm that it tends to avoid dense forests and to occupy edges and agricultural land around the forests. This in turn is likely to aggravate human–Blue bull conflict in future. Thus, proper conflict mitigation strategies should be adopted, and earlier studies have suggested that the cooperative guarding of crops during the harvesting season, changing cropping patterns, using (olfactory, acoustic, and visual) repellents, fencing, translocation, physical barriers, and sterilization could be beneficial to mitigate crop loss from the Blue bull and reduce retaliatory killing. 

## Figures and Tables

**Table 1:** A complete list of environmental variables compiled for this study. Environmental variables used for habitat suitability modeling for the Blue bull in Nepal are indicated by bold letters.

Source	Category	Variable	Unit
WorldClim	Bioclimatic	BIO1 = Annual Mean Temperature	°C
		**BIO2 = Mean Diurnal Range (Mean of monthly (max temp–min temp))**	°C
		**BIO3 = Isothermality (BIO2/BIO7) (×100)**	%
		BIO4 = Temperature Seasonality (standard deviation × 100)	°C
		BIO5 = Max Temperature of Warmest Month	°C
		BIO6 = Min Temperature of Coldest Month	°C
		BIO7 = Temperature Annual Range (BIO5-BIO6)	°C
		BIO8 = Mean Temperature of Wettest Quarter	°C
		BIO9 = Mean Temperature of Driest Quarter	°C
		BIO10 = Mean Temperature of Warmest Quarter	°C
		BIO11 = Mean Temperature of Coldest Quarter	°C
		BIO12 = Annual Precipitation	mm
		BIO13 = Precipitation of Wettest Month	mm
		**BIO14 = Precipitation of Driest Month**	mm
		**BIO15 = Precipitation Seasonality (Coefficient of Variation)**	%
		BIO16 = Precipitation of Wettest Quarter	mm
		BIO17 = Precipitation of Driest Quarter	mm
		**BIO18 = Precipitation of Warmest Quarter**	mm
		**BIO19 = Precipitation of Coldest Quarter**	mm
USGS	Topographic	Elevation	km
		**Aspect**	Degree
		**Slope**	Degree
GEOFABRIK		**Distance to water**	km
Landsat	Vegetation-related	Mean EVI, Minimum EVI, Maximum EVI (Enhanced Vegetation Index)	Dimensionless
GFC		**Forest**	Dimensionless
Department of Survey, Nepal	Anthropogenic	**Distance to settlement**	km
GEOFABRIK		**Distance to the motor road**	km
		**Distance to path**	km
HUMDATA		**Population density**	Dimensionless
		**Livestock density**	Dimensionless
ICIMOD		LULC	km

**Table 2:** The significant protected areas and areas of predicted suitable habitats for the Blue bull in Nepal.

Protected Areas (PAs)		Total Area (Km^2^)	Total Suitable Area (Km^2^)	Area (%) of Suitable Habitat Out of the Total Area of Specific PA	Area Covered within Specific PA Out of the Total Suitable Area within PA
Shuklaphanta	National Park	305	270.64	89%	8%
	Buffer Zone	243	228.78	94%	7%
Bardia	National Park	968	152.84	16%	5%
	Buffer Zone	327	162.67	50%	5%
Banke	National Park	550	111.29	20%	3%
	Buffer Zone	344	149.59	43%	5%
Chitwan	National Park	952	873.75	91%	27%
	Buffer Zone	750	518.83	69%	16%
Parsa	National Park	499	264.26	53%	8%
	Buffer Zone	298	51.14	17%	2%
Koshi Tappu	Wildlife Reserve	175	150.12	86%	5%
	Buffer Zone	173	173	100%	5%
Annapurna	Conservation Area	7629	34.63	0%	Less than 1
Shivapuri	National Park	159	26.23	16%	Less than 1
Black buck	Conservation Area	16	12.68	79%	Less than 1

**Table 3:** Percentage contributions of environmental variables to the model.

Environmental Variables	Generalized Linear Model	MAXENT.Phillips.2	Random Forest	Ensemble	Percentage Contribution
Slope	0.598	0.71	0.142	0.483	31%
Aspect	0.075	0.019	0.005	0.033	2%
Mean diurnal range	0	0	0.023	0.008	0%
Isothermality	0.212	0.043	0.005	0.087	6%
Precipitation of driest month	0.209	0.102	0.021	0.111	7%
Precipitation seasonality	0.492	0.027	0.03	0.183	12%
Precipitation of warmest quarter	0.202	0.042	0.015	0.086	6%
Precipitation of coldest quarter	0.095	0.049	0.045	0.063	4%
Distance to path	0.252	0.122	0.002	0.125	8%
Distance to the motor road	0.065	0	0.003	0.023	1%
Distance to settlement	0.14	0.072	0.014	0.075	5%
Distance to water	0.086	0.004	0.006	0.032	2%
Forest	0.128	0.159	0.04	0.109	7%
Population density	0.048	0.162	0.052	0.087	6%
Livestock density	0.086	0.007	0.019	0.037	2%

## Data Availability

The data that support the findings of this study are available from the first author, [B.D.], and second author, [A.B.], upon reasonable request.
